# A Multifunctional Graphene Oxide Platform for Targeting Cancer

**DOI:** 10.3390/cancers11060753

**Published:** 2019-05-29

**Authors:** Nikola Bugárová, Zdenko Špitálsky, Matej Mičušík, Michal Bodík, Peter Šiffalovič, Martina Koneracká, Vlasta Závišová, Martina Kubovčíková, Ivana Kajanová, Miriam Zaťovičová, Silvia Pastoreková, Miroslav Šlouf, Eva Majková, Mária Omastová

**Affiliations:** 1 Polymer Institute, SAS, Dúbravská cesta 9, 845 41 Bratislava, Slovakia; zdeno.spitalsky@savba.sk (Z.Š.); matej.micusik@savba.sk (M.M.); 2 Institute of Physics, SAS, Dúbravská cesta 9, 845 11 Bratislava, Slovakia; michal.bodik@savba.sk (M.B.); peter.siffalovic@savba.sk (P.Š.); eva.majkova@savba.sk (E.M.); 3 Institute of Experimental Physics, SAS, Watsonova 47, 040 01 Košice, Slovakia; konerack@saske.sk (M.K.); zavisova@saske.sk (V.Z.); kubovcikova@saske.sk (M.K.); 4 Institute of Virology, Biomedical Research Center, SAS, Dúbravská cesta 9, 845 11 Bratislava, Slovakia; viruivvi@savba.sk (I.K.); viruzato@savba.sk (M.Z.); virusipa@savba.sk (S.P.); 5 Institute of Macromolecular Chemistry AS CR, Heyrovského nám. 2, 162 06 Prague 6, Czech Republic; slouf@imc.cas.cz

**Keywords:** graphene oxide, magnetic nanoparticles, monoclonal antibodies, tumor targeting

## Abstract

Diagnosis of oncological diseases remains at the forefront of current medical research. Carbonic Anhydrase IX (CA IX) is a cell surface hypoxia-inducible enzyme functionally involved in adaptation to acidosis that is expressed in aggressive tumors; hence, it can be used as a tumor biomarker. Herein, we propose a nanoscale graphene oxide (GO) platform functionalized with magnetic nanoparticles and a monoclonal antibody specific to the CA IX marker. The GO platforms were prepared by a modified Hummers and Offeman method from exfoliated graphite after several centrifugation and ultrasonication cycles. The magnetic nanoparticles were prepared by a chemical precipitation method and subsequently modified. Basic characterization of GO, such as the degree of oxidation, nanoparticle size and exfoliation, were determined by physical and chemical analysis, including X-ray photoelectron spectroscopy (XPS), transmission electron microscopy (TEM), energy dispersive X-ray analysis (EDX), and atomic force microscopy (AFM). In addition, the size and properties of the poly-L-lysine-modified magnetic nanoparticles were characterized. The antibody specific to CA IX was linked via an amidic bond to the poly-L-lysine modified magnetic nanoparticles, which were conjugated to GO platform again via an amidic bond. The prepared GO-based platform with magnetic nanoparticles combined with a biosensing antibody element was used for a hypoxic cancer cell targeting study based on immunofluorescence.

## 1. Introduction

Recently, carbon-based nanoparticles have attracted a great amount of attention due to their interesting surface properties and ability to act as a platform for modification with various substances, including inorganic nanoparticles, drugs and ligands. Carbon nanotubes, quantum dots [1], nanoporous graphene [2], but mainly graphene and derivative graphene oxide [3], have frequently been used as new tools for targeting cancer. Graphene, with its numerous exceptional properties, has a wide range of applications in different fields, as has been described by a number of research groups [4,5,6,7,8,9]. Graphene, as a single atomic layer of graphite, was the first available two-dimensional crystal, with a large surface area and delocalized π electrons, and is hydrophobic, lipophilic [10] and a good electrical and thermal conductor [3,11,12]. Oxidized graphene, i.e., graphene oxide (GO), can be prepared in large quantities [13] through oxidation and exfoliation of bulk graphite, e.g., by a modified Hummers method [14]. GO, which is covered by oxygen functional groups, is hydrophilic, and the oxygen functional groups ensure its reactivity and covalent surface modification. GO contains oxygenated aliphatic regions (–OH) and (–O–) located above and below each layer, while the carboxylic groups are usually located at the edges of GO sheets. The hydrophilicity of GO is a useful property in bioapplications, and numerous different functionalizations with various molecules have been reported [15,16] for the use of modified GO in biosensors, drug carriers or tissue engineering. However, we should not forget about the possible cytotoxicity of GO, which was mentioned in vivo [17] and somewhat more in in vitro applications. Factors affecting the cytotoxic effects of GO have been described, such as the lateral dimension, the thickness of the nanolayers, the functional group, and especially the concentration [7,18,19,20].

In particular, the hydrophilic character of GO permits the production of reliable, highly sensitive and ultrafast targeted platforms. The size tunability of GO nanosheets from several microns down to less than 100 nm is also important for preparing GO nanosheets that are small enough to penetrate biological barriers, including those of tumor tissue [18,21]. This type of nanoparticle also enables the combination of diagnosis with therapy (theranostics) [22,23,24]. Photothermal therapy with a combination of treatments has resulted from the functionalization of GO with inorganic nanoparticles, other nanostructures or polymers to create composites [25,26]. The most widespread and facile method of functionalization is amidation. Shan et al. prepared modified GO with poly-L-lysine (PLL) through a covalent amide bond. The same bond used for functionalization of the gold electrode by PLL-functionalized GO [27]. The carboxylic acid groups (–COOH) of GO can interact with the amine groups of the nanostructures or nanoparticles that are being attached to create amide bonds. This process occurs in two steps in the presence of activators, namely, either 1-ethyl-3-(3-dimethylaminopropyl) carbodiimide (EDC) or N–ethyl–N’–(3–dimethylaminopropyl) carbodiimide hydrochloride (EDAC) and N–hydroxysuccinimide (NHS) [27,28,29]. The first step involves activation of the carboxyl group of GO and the formation of a stable active ester. The second step is a reaction between the active ester and an amine group on the attaching nanostructure, forming a covalent bond between GO and the nanoparticle [30]. Another important modification method is esterification. The esterification reaction involves a reaction between the –COOH groups localized on the edges of GO and CH_2_OH–terminated functional groups. For example, the grafting of poly(3-hexylthiophene) (P3HT) onto the GO surface was performed by esterification [31].

The key role of a GO-linked modifier is its selective transportation to the target cell or tissue, where the compound can be preferentially administered. Spherical magnetite nanoparticles (MNps) prepared by a coprecipitation method with ferric and ferrous salts in an alkaline aqueous medium [32] have great potential for use in oncological medicine due to their biocompatibility and facile synthesis. They can easily be tuned and functionalized for specific applications [30,33,34,35]. MNps have been coated by an amino acid polymer, poly-L-lysine, to improve their biocompatibility [36,37]. The importance of the amino acid L-lysine lies in the fact that it helps in the production of antibodies, is utilized by numerous hormones and enzymes and is necessary for tissue repair.

The bioconjugation of nanoparticles to proteins is critically important for a number of applications in life science research, diagnostics, and therapeutics [38]. Antibody molecules possess a number of functional groups suitable for modification or conjugation purposes. Monoclonal antibodies (MAb), antibodies made by identical immune cells that are all clones of a unique parent cell, are able to react specifically with tumor-associated antigens [39]. Consequently, the presence of MAb can increase the targeting properties of graphene oxide platforms. In particular, MAb M75 recognizes a linear epitope region of the enzyme carbonic anhydrase IX (CA IX). This enzyme is frequently expressed in human carcinomas because of its strong induction by tumor hypoxia, and it is absent from the corresponding normal tissue [40,41]. CA IX facilitates survival of cancer cells in hostile tumor microenvironment and contributes to their metastatic propensity. Due to its epitope specificity to CA IX, MAb M75 does not exhibit any cross-reactivity with other known members of the carbonic anhydrase family, which all lack similar regions [42]. Antibody-labeled GO has been used to detect cancer or, more generally, to detect proteins. An antibody-GO complex was used as a recognition element, which enabled rapid screening of aflatoxin B_1_ (AFB_1_) in a lateral flow system; the anti-AFB_1_ monoclonal antibody was prepared by Yu et al. [43]. Another study demonstrated the modification of screen-printed carbon electrodes by assembling graphene oxide to obtain electrochemical immunosensors with immobilized antibodies specific to mucin 1, which is a well-known tumor marker for a variety of malignant tumors [44]. Efforts to detect or target CA IX have been mostly focused on development of diverse nanoparticles decorated by inhibitors of Carbonic anhydrases with modifications conferring selectivity towards CA IX and/or other cell surface isoforms [45,46,47,48]. These approaches require access to active site of the enzyme, whereas the antibodies can bind CA IX even in an inactive state. In vitro tests were conducted at this stage of the research. Here we demonstrate that our approach, based on GO platforms decorated with MNps and M75 MAb can selectively bind cells expressing CA IX and thus can represent a promising tool for cancer imaging.

In this work, the preparation of a new type of GO-based platform containing magnetic nanoparticles and monoclonal antibodies is reported, with the aim of creating nanocarriers that are able to target hypoxia-inducing cancer cells. The combination of a MAb specific to CA IX and GO as a nanocarrier combined with magnetic nanoparticles is a novel and original approach for targeting CA IX-positive cancer cells. Magnetic nanoparticles immobilized with the MAb M75 were used and bound to the GO platform. These antibodies, which are specific to the hypoxia-induced extracellular enzyme CA IX, are one of the best intrinsic markers. The topology of the produced GO platform was examined using transmission electron microscopy (TEM) and atomic force microscopy (AFM), while its structure and surface properties were confirmed by X-ray photoelectron spectroscopy (XPS). AFM is a suitable technique rapidly screening the attached nanoparticles. As a result, the GO functionalization with poly-L-lysine-modified magnetic nanoparticles functionalized with MAb was validated by AFM. Additionally, TEM, immunofluorescence, viability, and flow cytometry tests were performed on the functionalized GO platforms.

## 2. Results

For our study, it was necessary to prepare GO platforms in the form of monolayers with particle sizes below 500 nm for further functionalization. The GO platforms were prepared by a modified Hummer and Offerman reaction. Obtained GO particles were washed with deionized water and hydrogen peroxide led to a brown suspension, and multiple cycles alternating between centrifugation and sonication were utilized. The centrifugation speed, duration, and number of cycles enabled the selection of different size distributions of the GO platforms. The purity and degree of GO oxidation were characterized by XPS method, while the morphologies and particle size of the GO platforms were examined by AFM and TEM.

### 2.1. Preparation and Functionalization of Graphene Oxide

**Figure 1:**
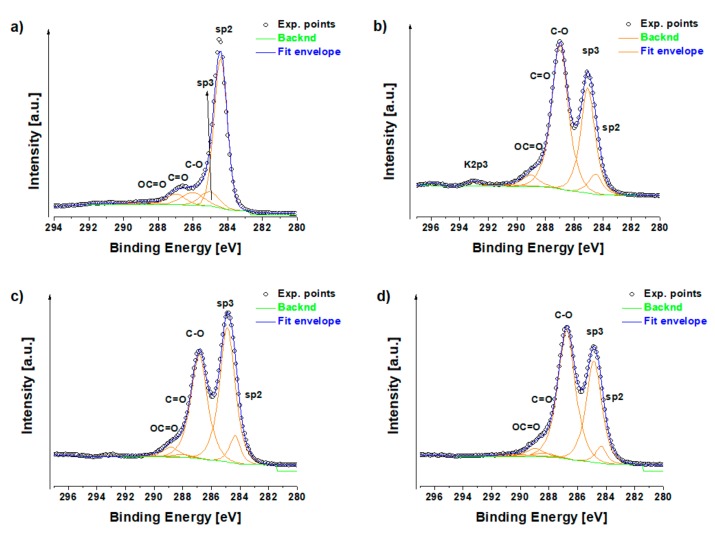
X-ray photoelectron spectroscopy C1s region spectra of (**a**) graphite powder; (**b**) graphene oxide; (**c**) graphene oxide platforms with magnetic nanoparticles modified antibodies (GO-MNps-MAb) and (**d**) graphene oxide platforms with magnetic nanoparticles modified antibodies in the presence of 1–ethyl–3–(3–dimethylaminopropyl) carbodiimide (GO-MNps-EDC-MAb).

X-ray photoelectron spectroscopy (XPS) was used to confirm the structural and chemical composition of the prepared graphene oxide platforms. First, the original graphite powder (G5) used for GO production was characterized. G5 showed only slight oxidation with no other contaminates on its surface (Table 1). GO product showed some traces of surface contamination by sulfur, potassium and chloride from the reaction procedure. Aluminum from the substrates was also detected since the samples for XPS measurements were prepared by drop casting a GO solution onto aluminum foil. Figure 1b clearly demonstrates the formation of carbon-oxygen bonds (C–O at ca 287 eV, C = O at ca 288 eV and OC = O at ca 289 eV) on the surface of the prepared GO, which was accessible for further modification. After modification with MNps and MAb C1s spectra of GO-MNps-MAb and GO-EDC-MNps-MAb are not differ much from the original GO, which indicates that the surface of GO is not fully covered. This could be very important for further potential application as drug delivery since there is still space to attach various active substances on the GO platform.

**Figure 2:**
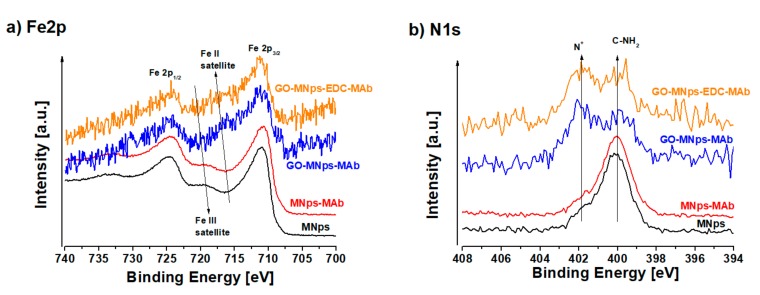
X-ray photoelectron spectroscopy of (**a**) Fe2p region and (**b**) of N1s region of magnetic nanoparticles (MNps), magnetic nanoparticles modified antibodies (MNps-MAb), graphene oxide platforms with magnetic nanoparticles modified antibodies (GO-MNps-MAb) and graphene oxide platforms with magnetic nanoparticles modified antibodies in the presence of 1–ethyl–3–(3–dimethylaminopropyl) carbodiimide (GO-MNps-EDC-MAb) (spectra are autoscaled).

We have discussed the surface chemistry of modified iron nanoparticles in a previous paper [36]. Fe_3_O_4_ can be written as Fe_2_O_3_:FeO (Fe III: Fe II) in a 2:1 ratio and exhibits contributions of these two iron valence states [49]. Fe_2_O_3_ possesses satellite features at ca. 719 eV and FeO at ca. 716 eV. In Fe_3_O_4_ the satellite feature is not visibly resolved because of the contributions of both of them (Fe III, Fe II) [50]. Figure 2a shows the comparison of the Fe2p region of MNps (Fe_3_O_4_-PLL), MNps-MAb, GO-MNps-MAb and GO-EDC-MNps-MAb. In the case of MNps there is a clearly visible Fe III satellite peak at ca. 719 eV, what indicates a Fe^3+^-rich surface. After MAb modification the Fe2p spectrum remained almost the same, with a similar Fe^3+^-rich surface. This implies that the MAb is binding through the PLL on the surface of MNps. When attached to the surface of GO, Fe2p peaks exhibit in both cases (GO-MNps-MAb and GO-EDC-MNps-MAb) a strong signal at ca. 716 eV corresponding to the Fe II satellite. This indicates that MNps interact with GO platform through the iron oxide nanoparticles and that Fe^3+^ is interacting with GO resulting in a partial reduction of iron. As it was shown in [36] iron oxide nanoparticles are most probably interacting with the carboxylate groups of GO leading to the chemisorption of MNps onto the GO surface.

In Figure 2b showing the N1s spectra, the detected signal at ca. 400 eV corresponds to C-N bonds like C-N-, C-NH-, C-NH_2_ and the one at ca. 402 eV it corresponds to some quaternized nitrogen labeled as N^+^ (XPS knowledge database of Avantage 5.9911, Thermo Fisher Scientific, Loughborough, UK). In the case of MNps it is a confirmation of PLL on the surface, there is almost no change after MAb attachment, but after attachment to the GO platform C–N and N^+^ with some proportional increase of N^+^ is still detected, indicating that the active NH_3_^+^ group of M75 is accessible on the surface.

**Figure 3:**
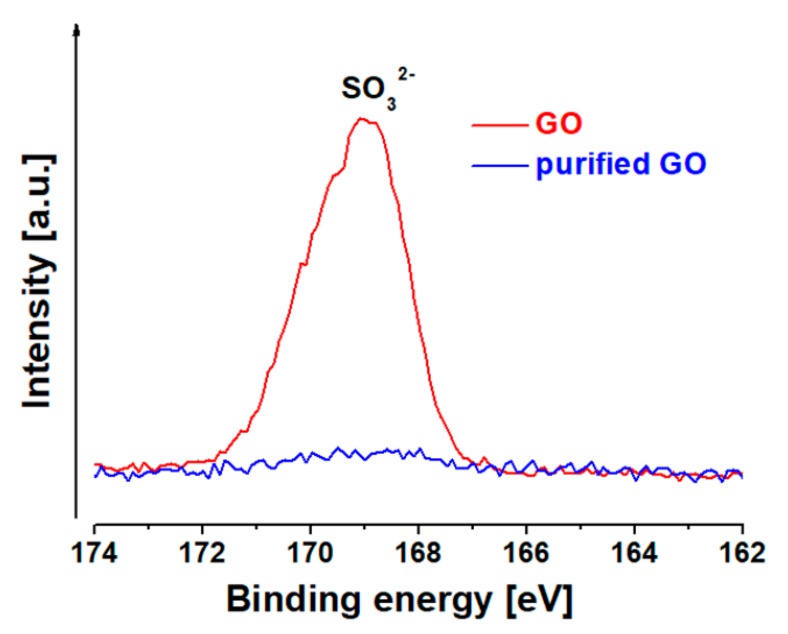
X-ray photoelectron spectroscopy S2p region spectra of graphene oxide (red) and purified graphene oxide (blue).

After this surface chemistry study, we decided to try additional purification of the initial GO platform and get rid of the relatively high (ca 2 at. %) amount of sulfur (S2p peak centered at 168.9 eV corresponding to SO_3_). This contamination comes from the sulfuric acid used during the GO preparation process. The presence of SO_3_ could be detrimental for the biocompatibility [10]; therefore, in the next step, this contaminant was successfully removed. After further purification with H_2_O_2_ and deionized water, the sulfur content decreased from 1.9 to 0.2 at. % (Table 1), as is obvious from comparison of the S2p peaks in Figure 3. For further reactions this purified GO was used.

**Figure 4:**
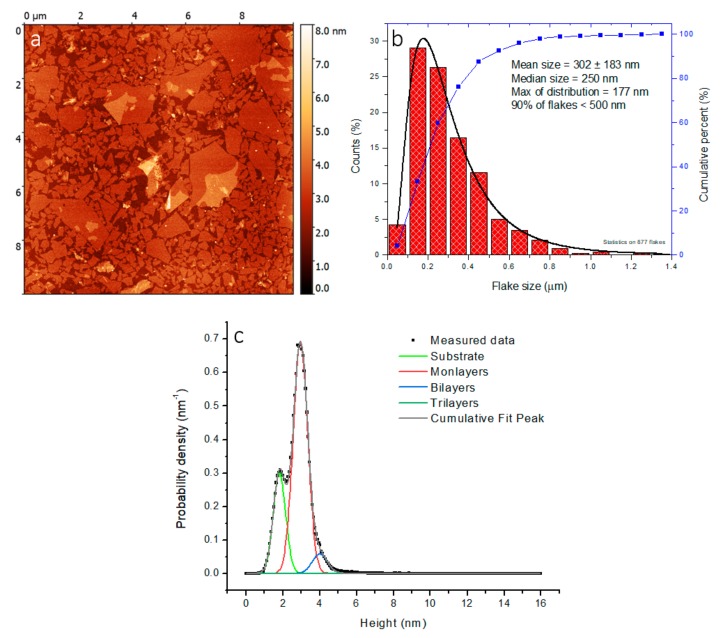
Purified graphene oxide: (**a**) atomic force microscopy (AFM) image, (**b**) size distribution of the graphene oxide platforms and (**c**) height analysis of AFM scan. The analysis of AFM scans was done by program Gwyddion [52] The size distribution was obtained manually.

The samples for AFM measurements were deposited by a modified Langmuir–Schaefer deposition method to obtain a thin monolayer film suitable for imaging. The preparation of a GO monolayer was described in our previous article [51,52]. A representative AFM image of the GO monolayer deposited on the Si substrate and the corresponding size distribution of the GO platforms are shown in Figure 4a,b, respectively. The prepared solution of GO was composed of approximately 90% GO monolayer platforms. The size distribution confirmed that 90% of all GO platforms had a lateral size smaller than 500 nm. The average lateral size of the GO platforms was 302 ± 183 nm. The heights analyzing of the AFM scan in Figure 4a is depicted in Figure 4c, showing the amount of monolayers and multilayers. The first peak at 1.81 nm with a peak width 0.67 nm corresponds to the substrate roughness value. The peak of monolayers is located at 2.95 nm with a peak width of 0.78 nm. The bilayers are detected at 4 nm with a width of 0.82 nm, so they are again 1 nm higher. The amount of trilayers is very small. Peak area for monolayers is 0.67863 nm^2^ and 0.05997 nm^2^ for bilayers, respectively. This analysis indicated that monolayers represent 91.8% in the prepared unmodified GO solution.

The stability of purified GO was examined by Zeta potential, with the results shown in Table 2. The general dividing line between a stable and non-stable suspension is usually taken as 30 or −30 mV and particles that have zeta potentials outside these limits are normally considered stable. According to values above ±30 mV, the sample is considered stable [53,54]. In the case of GO prepared by us, it is stable in the pH range of 4–11.

The GO prepared and purified of the sulfur residues was modified by MNps in the next step. The reaction described in the experimental section formed amide bonds between the hydroxyl groups of the GO surface and the amine groups of the poly-L-lysine shell on the MNps in the presence of EDC and sulfo-NHS. Hermanson has extensively reviewed the use of zero-length cross-linkers, to which EDC, NHS, and sulfo-NHS belong [55]. Their universal application to the conjugation of nanoparticles and biological substances has widely been used in a variety of systems. The conjugation of the GO platforms containing carboxylate groups with the MNps containing amine groups was thoroughly examined. Several reaction pathways were followed to determine the optimum ratio of GO, MNps, EDC and sulfo-NHS. Generally, EDC reacts with the carboxylates on the GO surface to form O-acylisourea as an intermediate reactive ester. The active EDC ester reacts with sulfo-NHS to form a sulfo-NHS ester intermediate, which rapidly reacts with the amines located on the MNps. We chose sulfo-NHS due to its hydrophilic properties and better reaction yield compared to that for the EDC linker alone [55].

**Figure 5:**
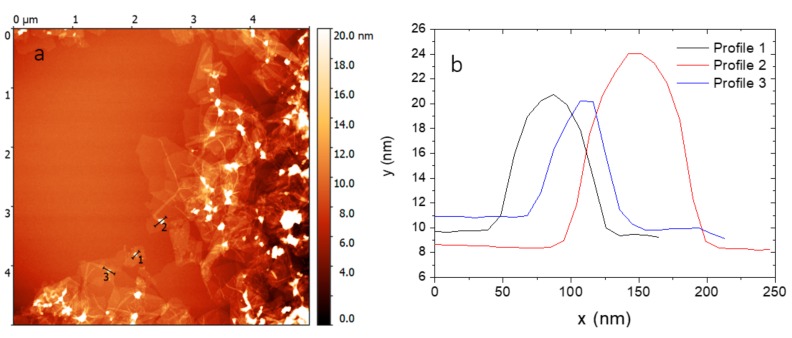
Modified graphene oxide platforms by magnetic nanoparticles: (**a**) atomic force microscopy (AFM) image, and (**b**) selected height profiles across the magnetic nanoparticles.

Initial reactions to verify the correct ratio of the binding linkers to GO as well as the ratio of GO to nanoparticles were performed on poly-L-lysine modified MNps. The ratio of GO:MNps was varied from 1:1 to 10:1. The formation of amide bonds between the hydroxyl groups of GO and the amino groups of poly-L-lysine on the MNps ensured that only unbound platforms were removed by washing with deionized water, while a magnet was set up. Bound MNps on the GO remained loosely dispersed in solution after washing. AFM was used for screening the GO surface modification with the MNps. At a high 1:1 ratio of GO:MNps, large MNps agglomerates were found on the GO surface. At a ratio of 10:1, a number of the GO platforms remained unmodified in suspension. Experimentally, the optimal ratio was 3:1. Figure 5 shows the evidence for the MNps on the GO platforms on the Si substrate for the abovementioned ratio of 3:1. The same ratio of 3:1 was used to functionalize the GO platform with MNps modified with MAb. The MNps modified with MAb were prepared in solution with and without the EDC binding agent.

### 2.2. Synthesis of Modified GO with MNps and MAb

**Figure 6:**
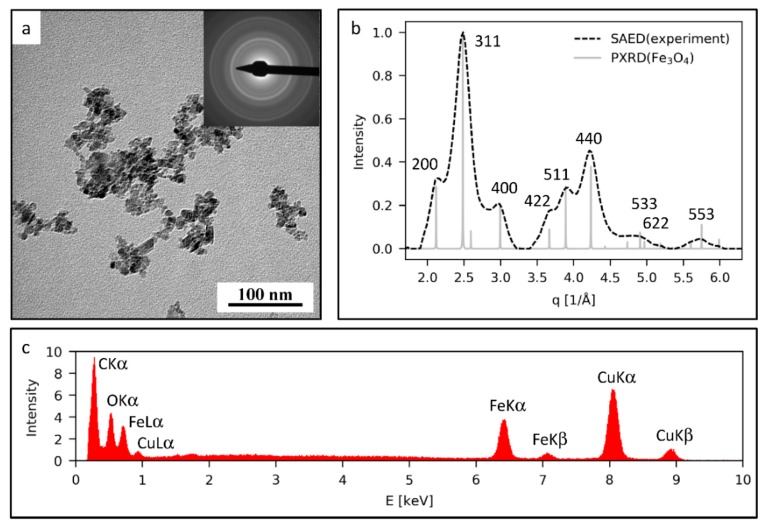
Transmission electron microscopy (TEM) analysis of the prepared magnetic nanoparticles (MNps): (**a**) TEM micrograph with corresponding selected area electron diffraction pattern (TEM/SAED) diffraction pattern, (**b**) comparison of the experimental TEM/SAED diffraction pattern with the theoretically calculated diffraction pattern of magnetite and (**c**) the energy dispersive X-ray analysis (TEM/EDX) spectrum of the MNps.

The GO functionalized platform was studied by TEM, but the initial elemental composition and crystalline structure of the prepared MNps were verified by the same method. The particles were visualized by bright field imaging (Figure 6a). Their selected area electron diffraction pattern (SAED, Figure 6a, upper left corner) was converted into a 1D-pattern and compared with the calculated powder X-ray diffraction pattern of magnetite (PXRD, Figure 6b). The perfect agreement between the experimental SAED pattern and the theoretical magnetite PXRD pattern proved that the prepared nanoparticles exhibited a magnetite structure (Figure 6b). This was further confirmed by the energy dispersive X-ray analysis (EDX) spectra (Figure 6c), which contained only the peaks corresponding to the supporting carbon-coated copper grid (peaks of C and Cu) and the peaks corresponding to the prepared iron oxide nanoparticles (peaks of Fe and O).

**Figure 7:**
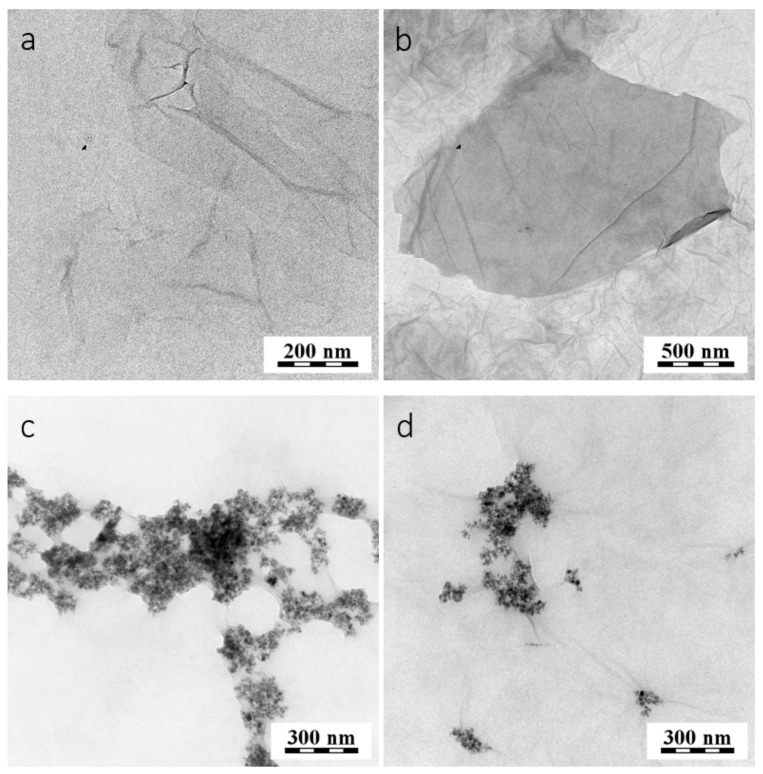
Transmission electron microscopy (TEM) micrographs of the (**a**,**b**) pure GO platforms; (**c**) graphene oxide platforms with magnetic nanoparticles modified antibodies (GO-MNps-MAb), and (**d**) graphene oxide platforms with magnetic nanoparticles modified antibodies in the presence of 1–ethyl–3–(3–dimethylaminopropyl) carbodiimide (GO-MNps-EDC-MAb).

The morphology of purified GO was examined by transmission electron microscopy (TEM). The representative micrographs of the purified GO in Figure 7a,b show a smooth platform surface with occasional folds. Functionalization of the platforms by magnetic nanoparticles could be clearly observed in the TEM images due to the high contrast of the attached nanoparticles, as illustrated in Figure 7c,d. Notably, some of the visualized platforms were quite large, with sizes >500 nm. The existence of these large platforms was in agreement with the AFM results (Figure 4). However, the majority of the nanoparticles exhibited dimensions well below 500 nm.

Magnetic nanoparticles with MAbs were prepared with and without the EDC coupling agent. The sample of conjugated nanoparticles prepared by reaction with EDC was labeled MNps-EDC-MAb, and the sample without the EDC binding agent was labeled MNps-MAb. The hydrodynamic size (100 nm) of the MNps without the MAbs can serve as a guide for locating the MNps. During GO modification, aggregates of the MNps were formed. In Figure 6c, comparatively more aggregates were present when compared with the platform shown in Figure 6d. Spherical MNps with a diameter of approximately 30–40 nm after being coating with poly-L-lysine resulted in smaller aggregates with fewer particles only in the case of GO-MNps-EDC-MAb. Notably, the MNps with MAbs were bound to the GO surface and remained attached to the surface even after several purification reactions.

### 2.3. Cell Viability Assay

Before all experiments with conjugation of magnetic nanoparticles and monoclonal antibodies to GO were done, a viability assay of cell lines in the environment with GO was performed during 5 days, as described in previous work [56], where application of GO as a low-toxicity platform for the next functionalization and was demonstrated.

**Figure 8:**
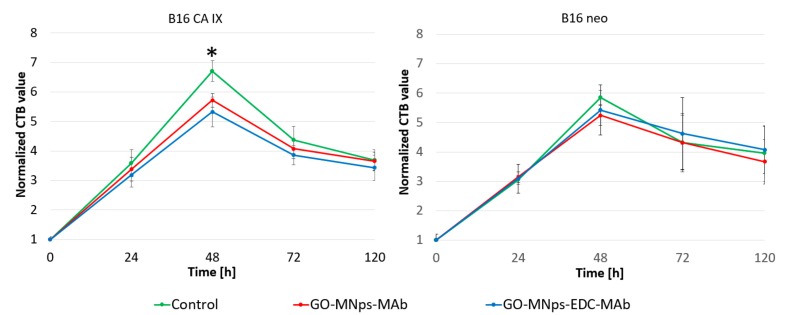
Viability testing of graphene oxide platforms with magnetic nanoparticles modified antibodies (GO-MNps-MAb) and graphene oxide platforms with magnetic nanoparticles modified antibodies in the presence of 1–ethyl–3–(3–dimethylaminopropyl) carbodiimide (GO-MNps-EDC-MAb) was performed on mouse melanoma cells (B16). Cell line was permanently transfected with full-length carbonic anhydrase IX (CA IX) cDNA. Control cell line was used (neo) without transfected CA IX cDNA. A significant difference in viability of B16 CA IX cells between the control and samples after 48 h cultivation is marked by *.

To control the toxicity of the prepared GO-platform functionalized with MNps and MAbs specific to CA IX, viability tests were performed to estimate the cytotoxicity or cytostatic effects. Pure medium without GO-MNps-MAb and GO-MNps-EDC-MAb was used as a control for both cell type and each sample type. The cell line B16 CA IX was B16-F0 mouse melanoma cells permanently transfected with the full-length CA IX cDNA. The mock-transfected cells, meaning without the CA IX enzyme, is labelled as B16-neo. The charts in Figure 8 show the results obtained during the five-day toxicity measurement with standard deviations. All experiments were performed in triplicates. Effect of the GO-MNps-MAb and GO-MNps-EDC-MAb on cell viability of cancer cell line B16-F0 at various periods of incubation was evaluated by CellTiter-Blue assay (CTB, Promega, Madison, WI, USA). First measurement was performed immediately after the establishment of the cell culture. The CTB fluorescence value of the control cells measured at the start of the experiment was set as 1. The CTB values of treated B16-neo and B16 CA IX cells were normalized to the non-treated control. Fluorescence measurement was performed after incubating for 24, 48, 72, and 120 h.

According to the trends indicated by the controls in B16-neo cell line, there was no cytotoxicity from the GO-MNps-MAb and GO-MNps-EDC-MAb in comparison to the control sample. The apparent behavior similarity of B16-F0 cells with CA IX and B16-neo cells without CA IX enzyme is the implication that both cell types come from a single cell line. But there is slight cytostatic effect specific only to B16 cells with CA IX (B16 CA IX) from both functionalized GO platforms (GO-MNps-MAb and GO-MNps-EDC-MAb), detected with the reduced trend at the graph in comparison to the control sample. This was also confirmed by statistical analysis using the Student’s *t*-test, where a significant difference between control and samples was obtained for a B16 CA IX cells after 48 h. However, this finding can be seen as a positive result or added value. Prepared platforms have the task of specifically targeting cells, which is also indirectly confirmed in this case. By looking at the significant difference in cells with CA IX enzyme and we do not observe this difference for neo cells, it is possible to speak about specific uptake/binding of functionalized platform to cells even in viability assay.

### 2.4. Immunofluorescence Assay

The immunofluorescence assay can confirm the presence of modified GO platforms and bound magnetic nanoparticles with antibodies in cells. Besides that, the immunofluorescence indirectly validated the binding of the MNps with MAbs onto the GO. The GO itself is not able to produce the green fluorescence of a positive response. The positive signal was due to the secondary anti-mouse Alexa Fluor^®^ 488 antibody (Thermo Fisher Scientific, Loughborough, UK) bound to MAb M75. The MAb is specific to extracellular antigen CA IX, and free antibody M75 is not able to internalize into the cells at 37 °C [40].

**Figure 9:**
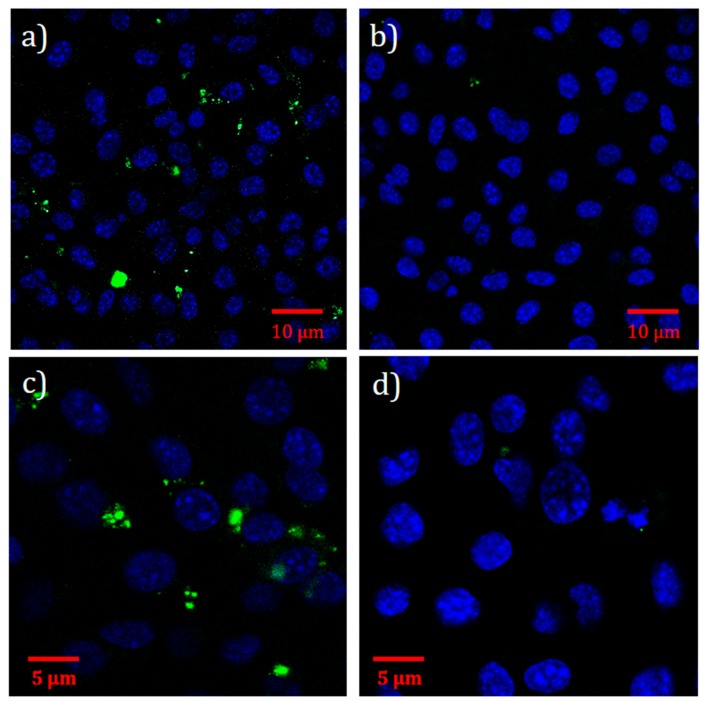
Immunofluorescence of graphene oxide platforms with magnetic nanoparticles modified antibodies (GO-MNps-MAb) into the B16 cells. Sample GO-MNps-MAb was examined in (**a**) B16 cells with carbonic anhydrase IX (CA IX) and (**b**) B16-neo cells without CA IX. Sample with the binding agent graphene oxide platforms with magnetic nanoparticles modified antibodies in the presence of 1–ethyl–3–(3–dimethylaminopropyl) carbodiimide (GO-MNps-EDC-MAb) were examined in the same cell types: (**c**) B16 CA IX and (**d**) B16-neo cells.

In order to verify the specific binding of the modified GO platform, the B16 CA IX cells and no enzyme B16-neo were used for the assay. Cells without enzyme CA IX represent the negative control. There were differences between the neo cells and the cells expressing CA IX. In the negative control (B16-neo cells) in either case of exposure to samples (GO-MNps-MAb and GO-MNps-EDC-MAb) with secondary antibody, no or only a minimal signal at 488 nm was observed. In contrast, B16 CA IX cells, contain sufficient secondary antibody signal in both cases (Figure 9a,c) around their nuclei. This signifies a specific binding of the primary antibody with magnetic nanoparticles bound to GO platform to cells with CA IX. For cells exposed to the GO-MNps-MAb sample in Figure 9a, green signal aggregates were observed at multiple places. In the case of the GO-MNps-EDC-MAb sample, smaller aggregates were observed and also smaller points characterizing individual modified GO platforms could be identified. For this reason, the GO-MNps-EDC-MAb sample is presented with a two-fold approach as shown in Figure 9c,d. The GO-MNps-EDC-MAb sample, which was prepared in solution with the binding agent EDC, showed more intense fluorescence. These results were supported by another experiment that quantified the binding of functionalized GO platforms to live cells with CA IX enzyme. Figure 9 shows a positive signal detected on the surface of as well as in the cells. This evidence for cellular internalization of functionalized GO platforms (GO-MNps-MAb and GO-MNps-EDC-MAb) indicated an interesting phenomenon. While the free antibodies could not penetrate the cell membranes, the antibodies conjugated to the MNps and GO could pass through the membranes and accumulate within the cells. The described phenomenon would be helpful in later studies on drug uptake in certain cell types and intracellular localization.

### 2.5. Flow Cytometry

Using flow cytometry it is possible to quantify the specific binding of functionalized GO platform to carbonic anhydrase IX. The measured cell line was MDCK line with (MDCK CA IX) and without (MDCK neo) CA IX enzyme. Table 3 presents results of binding for negative controls and samples. There were done two negative controls. Unmodified GO platforms in the first row and cells labeled only with secondary antibody anti-mouse Alexa Fluor^®^ 488 (marked as A488, Thermo Fisher Scientific, Loughborough, UK) in the second row of the table.

**Figure 10:**
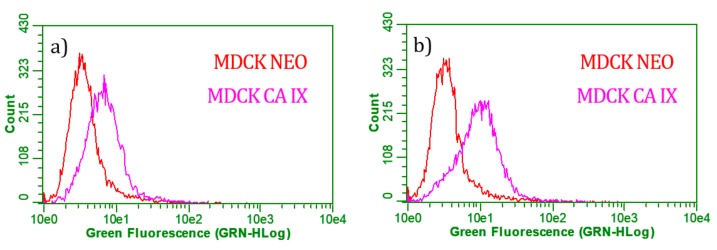
Cytometric analysis of (**a**) graphene oxide platforms with magnetic nanoparticles modified antibodies (GO-MNps-MAb) and (**b**) graphene oxide platforms with magnetic nanoparticles modified antibodies in the presence of 1–ethyl–3–(3–dimethylaminopropyl) carbodiimide (GO-MNps-EDC-MAb) samples.

As shown in line 2 in Table 3, the anti-mouse antibody alone did not demonstrate cell binding. From the results in row 1 in Table 3, there is proof of interaction between GO and fluorescent labeled anti-mouse antibody. If there is some low interaction between GO and secondary anti-mouse antibody Alexa Fluor^®^ 488, nevertheless it is not able to produce non-specific binding to any cell line, what is shown by low presence of GO as negative control in cells. The examined samples were functionalized GO platforms with magnetic nanoparticles modified antibodies, designated GO-MNps-MAb and GO-MNps-EDC-MAb. The difference between these samples is in the preparation of modified nanoparticles with monoclonal antibodies. In the GO-MNps-MAb sample, the EDC binding agent was not used for modification, as opposed to the GO-MNps-EDC-MAb sample, which has so far shown better results. Otherwise, it was not even after a cytometric analysis that confirmed these findings. In Figure 10, graphs comparing both samples. The GO-MNps-EDC-MAb sample is more positive for MDCK CA IX cells, which corresponds to the shift to right on x-axis of the magenta-stained peak for MDCK CA IX cells. Also, the 4th row of the table correlates with the graph where 48% of all living cells in population contained at least one functionalized platform. In the case of MDCK neo cells, only 6% of the live cells were positive and contained at least one platform. This corresponds to the specific binding of the functionalized GO to CA IX-positive cells.

The hydrophilic character and better reactivity caused by the oxygen-containing functional groups indicated the potential biological and medical use of GO in targeted therapy. Our study demonstrated the functionality of the prepared multifunctional GO platforms conjugated with MNps and MAb. The specificity of MAb to CA IX remained unchanged even after conjugation to the MNps, and the GO platform was targeted into the cancer cells. The results present a new possibility for the use of GO in cancer therapy with minimal side effects.

## 3. Discussion

Recent technological advances and new possibilities for the application of graphene-based sensors in biomedicine open up new possibilities for cancer detection. The intention was to use graphene oxide (GO) as a platform for developing a new type of a promising tool for cancer imaging. With the purpose of such functionalization, we have prepared GO platforms using a modified Hummer and Offerman reaction. A modified Hummer’s method was used for example, for the preparation of nanographene, which was after PEGylation was used for the delivery of the insoluble camptothecin (CPT) analogue, SN38 [19,57]. Graphene oxide platforms were modified by magnetic nanoparticles and monoclonal antibodies. For this study, it was necessary to prepare GO platforms in the form of monolayers with particle sizes below 500 nm for further functionalization. Seabra et al. have reviewed the behaviour of GO in terms of cytotoxicity [19]. They described the relationship with its thickness (number of layers), size, concentration, time of exposure, method of functionalization, and it also depends on the cell line on which the GO toxicity is investigated. More times the comparison of graphene, GO and reduced GO is mentioned, when GO has the least toxicity due to its hydrophilic character caused by functional groups [17,19]. Likewise, GO with a surface containing COOH groups and NH_2_ groups [58,59] have shown less toxicity than non-functionalized [19,57]. However, the GO toxicity phenomenon is still not sufficiently investigated and therefore the claims cannot be generalized. For this reason, we performed cytotoxicity tests on the B16-F0 cell line after functionalization, with no toxicity. GO modification with magnetic nanoparticles and a monoclonal antibody specific to the CA IX marker is new approach and combination of nanomaterials and antibody. The specificity of this MAbs for tumor cells plays an essential role in the preparation of a nanoparticle modified by magnetic nanoparticles and monoclonal antibodies. Differences between B16 CA IX cells and neo cells without CA IX enzyme confirm specific binding of functionalized GO platform to cancer marker cells. Targeting functionalized GO nanoparticles to tumor cells at this stage of research represents a vision of possibilities to visualize cancer cells in vitro for now and in vivo at later stages of research.

Rationale for the exploitation of CA IX as a target for cancer imaging stems from an increasing number of studies demonstrating that its expression pattern is tightly related to cancer. Meta-analysis of studies encompassing more than 24,000 patients revealed strongly significant associations between CA IX expression evaluated by immunohistochemistry and all endpoints: overall survival, disease-free survival, loco-regional control, disease-specific survival, metastasis-free survival, and progression-free survival [60]. Subgroup analyses showed similar associations in the majority of tumor sites and types. The results showed that patients having tumors with high CA IX expression have higher risk of disease progression, and development of metastases, independent of tumor type or site.

Actually, transcription of CA IX is strongly induced by hypoxia and the protein is also functionally involved in cancer progression due to regulation of pH, control of cell adhesion and invasion, facilitation of glycolytic metabolism and metastatic dissemination as supported by data from suppression of CA IX in diverse cancer models [61]. Since hypoxia as well as acidosis are microenvironmental stresses linked to aggressive cancer behavior, visualization of regions where cancer cells are exposed and adapted to these stresses offers opportunity to predict prognosis and therapy outcome. In this context, it is meaningful to use CA IX for targeting cancer via detection of hypoxic/acidic tumors. In addition to classical means of imaging tumors with CA IX via radiolabeled antibodies, the literature describes several nanoparticle-based approaches. Except one study using silica nanoparticles coated with the anti-CA IX MAb M75 [62], all other studies exploited carbonic anhydrase inhibitors. As CA inhibitors are principally acting towards diverse CA isoforms due to similarities in their active sites, selectivity to CA IX was conferred by diverse carriers [45], gold nanoparticles [46], pH-responsive nanoparticles [47], polymer-based photodynamic nanoinhibitor particles [48]. However, the approaches using inhibitors principally differ from the approach using monoclonal antibodies. Inhibitors are expected to access and bind to the catalytic site of the enzymatically active CA IX, whereas the MAb directed to the N-terminal region of CA IX, such as M75, can access and bind CA IX outside of the catalytic site and thus the binding does not depend on the activity of the enzyme and is highly specific. This might be particularly important in tumor tissues where CA IX is present on the surface of acutely hypoxic cells (due to induction by hypoxia) as well as of post-hypoxic and/or acidic cells (due to high protein stability) that already underwent adaptation to hypoxia and acidosis and have aggressive properties, thus broadening the clinically meaningful area of tumor targeting. The data presented here suggest that this approach is viable and deserves further development.

## 4. Experimental Section

### 4.1. Materials

Graphite (particle size 5 µm, SGL Carbon GmbH, Meitingen, Germany), 1–ethyl–3–(3–dimethylaminopropyl) carbodiimide (EDC, 98+%, ACROS Organics part of Thermo Fisher Scientific, Geel, Belgium), sulfo-NHS (N-hydroxysulfosuccinimide, 98+%, ACROS Organics part of Thermo Fisher Scientific, Geel, Belgium), phosphate buffered saline (PBS) tablets (100 mL, Biotechnology grade, VWR™, part of Avantor, Bridgeport, NJ, USA) and TWEEN^®^ 20 (Sigma-Aldrich Co., St. Louis, MO, USA) were used as received. All chemicals used for graphite oxidation were of 97% analytical grade purity and were used as received. Magnetic nanoparticles coated with poly-L-lysine (MNps), with a hydrodynamic size of 27–33 nm, and magnetic nanoparticles coated with poly-L-lysine and conjugated with monoclonal antibody M75 (MNps-MAb and MNps-EDC-MAb) were prepared and synthesized as described in a previous report [37].

### 4.2. Preparation of Graphene Oxide

Graphene oxide (GO) was prepared from graphite by a modified Hummers and Offeman method [14]. The exfoliation was performed by numerous repetitions of mixing, sonication and centrifugation with a Sigma 3–30 K centrifuge (Sigma Centrifuges, Newtown, UK) at 10,000× *g* for 40 min. The prepared solution had a GO concentration of approximately 10 mg/mL.

### 4.3. Modification of the Graphene Oxide Surface with Magnetic Nanoparticles

A PBS solution and TWEEN^®^ 20 (Sigma-Aldrich Co., St. Louis, MO, USA) were mixed with the GO solution. A solution of EDC and sulfo-NHS was added to the GO solution at a weight ratio of 1:1. A suspension of the magnetic nanoparticles coated with poly-L-lysine was added dropwise into the reaction mixture. The weight ratio of GO to MNps was 3:1. The reaction mixture was placed in an ultrasound bath for two hours. The GO platforms modified with MNps were separated in the reaction vessel with a magnet and purified several times with deionized water. The prepared platform was labelled GO-MNps.

### 4.4. Synthesis of GO-MNps-MAb and GO-MNps-EDC-MAb

Two kinds of MNps conjugated with MAb were employed for GO modification. The first one was prepared by reaction with a binding agent, EDC, and labeled MNps-EDC-MAb, the second, without the EDC binding agent, was labeled MNps-MAb. The detailed preparation procedure was described in a previous report [37].

In brief, magnetite nanoparticles were precipitated from an aqueous solution of Fe2+ and Fe3+ ions upon gradual addition of ammonium hydroxide. The formed precipitate was washed with ultrapure water and agitated with an immersed sonicator probe (Model 450, BRANSON, Danbury, CT, USA) for 5 min at a power of 280 W in a water bath. Then, the suspension of nanoparticles was mixed with a PLL solution (0.1%) at the theoretical PLL: Fe_3_O_4_ weight ratio of 1 and again sonicated in an ice bath. Next, the samples were ultracentrifuged at 44,000 g for 2 h at 4 °C. The sediment was carefully redispersed in ultrapure water and collected to form the PLL-MNps sample with magnetite and PLL concentrations of 18.5 and 3.6 mg/mL, respectively. The modified nanoparticles were subsequently incubated with an added MAb solution, with and without EDC, in PBS. After incubating for 24 h, the nanoparticles were centrifuged and washed to ensure that the unbound MAb was removed. The GO particles were later modified with both types of MNps as described in Section 2.3, and the samples were labeled GO-MNps-MAb and GO-MNps-EDC-MAb.

### 4.5. Characterization Techniques

AFM images of the GO platforms were obtained with a MultiMode 8 microscope (Bruker, Billerica, MA, USA). The measurements were performed in the ScanAsyst mode in air. The AFM tips used for the measurements were ScanAsyst-Air probes (Bruker, Billerica, MA, USA). The XPS measurements were performed using a Thermo Scientific K-Alpha XPS system (Thermo Fisher Scientific, Loughborough, UK) equipped with a microfocused monochromatic Al Kα X-ray source (1 486.6 eV). The Zeta potential (Malvern, Houston, TX USA) was measured at Zetasizer Nano-ZS for purified G0 in the pH range from 2 to 11. pH was adjusted with KOH solution and controlled by pH/mV/Temperature BENCH METER (Hanna Instruments Czech s.r.o., Prague, Czech Republic). TEM micrographs of the platforms were obtained with a Tecnai G2 Spirit Twin 12 transmission electron microscope (FEI, Prague, Czech Republic). A small droplet (2 μL) of a suspension containing the magnetic nanoparticles (concentration ca. 5 mg/mL) or the platforms with and/or without the nanoparticles (concentration ca. 1 mg/mL) was deposited onto a commercial carbon-coated copper grid for TEM and left to equilibrate (8 min). Then, the excess solution was removed by touching the bottom of the grid to a small piece of filter paper (the fast drying method, which minimizes possible drying artifacts). The dried samples were observed in the transmission electron microscope using bright field imaging at 120 kV. Moreover, the magnetic nanoparticles were also analyzed by energy dispersive analysis of X-rays (EDX, to confirm their elemental composition) and by selected area electron diffraction (SAED, to confirm their expected magnetite crystalline structure). The SAED diffraction pattern was compared with the calculated powder X-ray diffraction pattern (PXRD) of magnetite, as described in a previous report [63].

### 4.6. Cell Viability Assay

The cytotoxicity or cytostatic effect evaluation of the modified GO-MNps-MAb and GO-MNps-EDC-MAb was performed on B16 mouse melanoma cells permanently transfected with the full-length CA IX cDNA (B16 CA IX), and mock-transfected cells B16‑neo. The CellTiter-Blue (CTB) viability assay (Promega, Madison, WI, USA) was used. Testing was performed according to the instructions provided by the manufacturer. The cells were incubated in 96‑well plates for 24 h cell at 37 °C. The measurements were performed immediately after the establishment of the cell culture, and the following values were measured after 24, 48, 72 and 120 h. The CTB fluorescence value of the control cells measured at the start of the experiment was set as 1. The CTB values of treated cells were normalized to their non-treated control. Each measurement was performed after the direct addition of 20 μL of the CellTiter-Blue solution to the wells and incubation for 4 h at 37 °C. The fluorescence was recorded with a 530 nm/590 nm (excitation/emission) filter set using a Synergy HT microplate reader (Bio-Tek, Winooski, VT, USA). The samples were run in triplicate for each concentration of the GO-MNps-MAb and GO-MNps-EDC-MAb.

### 4.7. Immunofluorescence Assay

The B16 mouse melanoma cell line with (B16 CA IX) and without (B16‑neo) CA IX was plated (300,000 cells per Petri dish) on sterile glass coverslips 24 h before the experiment. The live cells were incubated at 4 °C for 30 min with GO-MNps-MAb and GO-MNps-EDC-MAb diluted in culture medium to characterize the uptake of MAb by the extracellular enzyme CA IX. The cells were then washed to remove unbound platforms and transferred to incubate at 37 °C for 3 h to allow endocytosis. The washing was repeated again to remove unbound GO nanoparticles and antibodies. The presence of conjugated M75 was visualized in cells fixed in ice-cold methanol at −20 °C for 5 min using an anti-mouse Alexa Fluor^®^ 488 (Thermo Fisher Scientific, Loughborough, UK) conjugated secondary antibody. Finally, the cells were mounted onto slides and analyzed by confocal laser-scanning microscopy (CLSM) on a Zeiss LSM 510 Meta instrument (Heidelberg, Germany).

### 4.8. Flow Cytometry Assay

Madin-Darby Canine Kidney cells with CAIX (MDCK CAIX) and without enzyme (MDCK neo) were used for cytometric analysis of the functionalized GO platform by magnetic nanoparticles and conjugated M75 antibodies. Cells were established at 35,000/cm2. GO platform samples were pre-incubated with anti-mouse Alexa Fluor^®^ 488 antibody diluted 1:1000, 1 h at 37 °C on an orbital stirrer. After the cells adhered to the surface of the plate, preincubated 1:4 GO platforms samples were added to the culture media. The cells were then incubated overnight at 37 °C. Subsequently, the culture media were removed; the cells were washed three times with PBS solution and released with trypsin. The cell suspensions were then washed with Verzene (cell centrifugation was 5 min at 400 g) and analyzed by Guava EasyCyte plus flow cytometry (Millipore, Burlington, MA, USA) using a 525/30 nm filter. Data was evaluated using Cytosoft 5.2 software (Millipore, Burlington, MA, USA) with Guava ExpressPro.

## 5. Conclusions

Graphene oxide platforms were prepared with a mean size of 302 ± 183 nm, as determined by AFM. The GO was purified to remove sulfur. The surface composition of the prepared GO was characterized by XPS; additionally, the oxidation state and ratio of C:O were determined by XPS. TEM micrographs that confirmed the smooth surface of the GO platforms and the attachment of the magnetic nanoparticle onto the GO surface. A ratio of 3:1 for GO:MNps was chosen for further functionalization of the GO by MAb. After performing toxicological assays at B16-F0 cell line, no cytotoxicity effect of the multifunctional GO platforms were ascertained. However, there was a significant cytostatic effect for B16 CA IX cells after 48 h. The immunofluorescence test indirectly confirmed the bonding between graphene oxide and the magnetic nanoparticles conjugated to the monoclonal antibody. The selectivity of the GO-MNps-MAb and GO-MNps-EDC-MAb platforms toward tumor cell targeting was demonstrated; therefore, the potential use of GO-MNps-EDC-MAb in tumor treatment was indicated. The GO-MNps-EDC-MAb sample prepared with an EDC binding agent showed better results in the immunofluorescence assay. As an initial step, this study provides promising evidence of tumor targeting with broad potential for visualization and future tumor treatment.

## Figures and Tables

**Table 1:** X-ray photoelectron spectroscopy analysis of graphite, GO and purified GO.

Sample		Surface Chemical Composition [at. %]				
	C1s	O1s	S2p	N1s	Fe2p	K 2s/Cl2p/Al2p/P2p/Si2s/Na1s/Br3d
Graphite G5	90.2	9.8	—	—	—	—/—/—/—/—/—/—
GO	61.7	33.7	1.9	0.9	—	0.5/0.4/1.0/—/—/—/—
MNPs	29.6	41.2	0.8	6.8	19.7	—/—/——/—/1.0/1.0
MNps-MAb	32.7	39.7	—	4.3	12.0	0.1/3.4/—/2.2/0.3/5.3/—
GO-MNps-MAb	69.2	26.6	0.6	1.0	0.2	—/0.6/1.4/—/0.1/0.3/—
GO-MNps-EDC-MAb	62.5	32.8	0.8	0.7	0.2	—/0.6/2.4/—/—/0.1/—
Purified GO	70.8	28.2	0.2	0.1	—	—/—/0.9/—/—/—/—

*XPS = X-ray photoelectron spectroscopy; G5 = Graphite powder; GO = graphene oxide; MNps = magnetic nanoparticles; MNps-MAb = magnetic nanoparticles modified antibodies; GO-MNps-MAb = graphene oxide platforms with magnetic nanoparticles modified antibodies; GO-MNps-EDC-MAb = graphene oxide platforms with magnetic nanoparticles modified antibodies in the presence of 1–ethyl–3–(3–dimethylaminopropyl) carbodiimide.

**Table 2:** Zeta potential analysis of purified graphene oxide.

pH	2	3	4	5	6,5	8	9	10	11
GO	−12	−15.8	−39.3	−35.3	−41.3	−46.3	−44.4	−42.8	−44.9

**Table 3:** Flow cytometry analysis of specific binding to CA IX cells.

No.	Sample	MDCK Cell Line	
		CA IX [%]	Neo [%]
1	GO, neg. control	1.12	1.43
2	A488, neg. control	0.10	0.03
3	GO-MNps-MAb	22.16	6.07
4	GO-MNps-EDC-MAb	48.63	6.01

MDCK = Madin-Darby Canine Kidney cells; CA IX = cells with carbonic anhydrase IX; Neo = cells without carbonic anhydrase IX; GO = graphene oxide; A488 = Alexa Fluor^®^ 488; GO-MNps-MAb = graphene oxide platforms with magnetic nanoparticles modified antibodies; GO-MNps-EDC-MAb = graphene oxide platforms with magnetic nanoparticles modified antibodies in the presence of 1–ethyl–3–(3–dimethylaminopropyl) carbodiimide.
